# Efficacy of Antenatal Treatment Using IVIG and Steroids in Managing Subsequent Pregnancy Following a Case of Neonatal Alloimmune Thrombocytopenia in Sibling

**DOI:** 10.1155/crpe/5244463

**Published:** 2026-09-24

**Authors:** Naheed Abedin, Ahmad Abdulkader, Tiffany Nguyen, Mansi Ranasaria

**Affiliations:** ^1^ Richmond University Medical Center, 355 Bard Ave, Staten Island 10310, New York, USA, rumcsi.org; ^2^ Nicklaus Children’s Hospital, Miami, Florida, USA, nicklauschildrens.org; ^3^ San Joaquin General Hospital, French Camp, Californi, USA; ^4^ University of Maryland, College Park, Maryland, USA, umaryland.edu

## Abstract

Neonatal alloimmune thrombocytopenia (NAIT) can present as severe thrombocytopenia in an otherwise healthy neonate and may lead to serious bleeding, intracranial hemorrhage, and even death. This report presents two siblings affected by NAIT. The first sibling was diagnosed when the infant presented after birth with petechial hemorrhages and a profoundly low platelet count. During the subsequent pregnancy, the mother was treated with a protocol of IVIG and steroid. This resulted in a higher platelet count at birth in the second sibling, although still severely thrombocytopenic, without physical signs or symptoms of thrombocytopenia. In this case, the infant responded rapidly to an antigen‐matched platelet transfusion. This case series calls attention to the importance of close follow‐up and optimum management of NAIT.

## 1. Introduction

Fetal neonatal alloimmune thrombocytopenia (FNAIT) also known as neonatal alloimmune thrombocytopenia (NAIT) is a major cause of thrombocytopenia in healthy term newborns, occurring with an incidence of 1 in 1000 to 2000 live births [1, 2]. Most cases of NAIT are mild but severe morbidity and mortality including intracranial hemorrhage (ICH) can be attributed to NAIT. It results from incompatibility of maternal alloantibodies against human platelet antigens (HPAs) present on the fetal platelets. HPA‐1a alloantibody is the most common cause of NAIT, with the incidence of HPA‐1a–negative (HPA‐1bb) phenotype in Caucasian population being about 2.5% [2].

NAIT is characterized by a platelet count < 150,000/mm^3^, with severe cases defined as < 25,000/mm^3^. Risk factors for worsening thrombocytopenia include subsequent pregnancies, increasing maternal HPA‐1a antibody levels, and fetal/neonatal ICH during previous pregnancy. Treatment is dependent on the severity of thrombocytopenia but can involve antigen‐negative platelet transfusion, intravenous immunoglobulin (IVIG), and/or corticosteroids [2, 3].

Antenatal management of subsequent pregnancies has ranged from serial fetal blood sampling (FBS) and intrauterine platelet transfusions (IUPT) to weekly maternal IVIG and corticosteroid treatments. FBS and IUPT have been associated with booster of alloimmunization, fetal bleeding complications, intrauterine death, and emergency caesarian sections. Noninvasive measures, such as IVIG and corticosteroids, are preferred. However, since severe NAIT is rare, studies have shown a variety of management regimens [4].

This series of cases outlines the clinical management and outcome of two siblings diagnosed with severe NAIT, with particular emphasis on the mother’s antenatal care during the second pregnancy. It underscores the important benefit of using both IVIG and steroids together for effective antenatal intervention in NAIT.

## 2. Case 1

A small for gestational age (SGA) newborn female was delivered by spontaneous vaginal delivery at 39 and 3 weeks of gestation with a birth weight of 2600 g to a 30‐year‐old Gravida 1 Para 0 mother. The pregnancy and delivery were uneventful with negative Group B Streptococcus (GBS) and prenatal serology results. The newborn had reassuring APGAR scores of 9 at 1 and 5 min after birth. However, upon examination, widespread petechiae were noted over the back, extremities, and torso. The newborn’s complete blood count was performed and revealed severe thrombocytopenia with a platelet count of 3000/mm^3^ at approximately 2 h of life. Given the severity of thrombocytopenia and the presence of petechiae, the newborn was promptly transferred to the neonatal intensive care unit (NICU) for further management.

**FIGURE 1:**
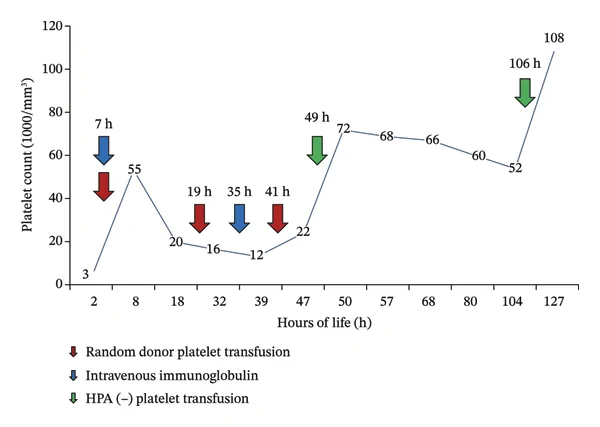
The first sibling’s platelet count throughout the NICU stay, relative to the different interventions. The red arrows indicate the administration of 3 units of random donor platelets, the blue arrows represent 2 doses of intravenous immunoglobulin, and the green arrows denote 2 HPA‐negative platelet transfusions.

Upon arrival to the NICU, the newborn received an initial transfusion of random donor platelets at a dose of 15 mL/kg, followed by an IVIG transfusion at a dose of 1 g/kg. This intervention resulted in an increase in platelet count to 55,000/mm^3^. However, the following day, the platelet count dropped to 12,000/mm^3^, prompting another transfusion of random donor platelets, which raised the count to 22,000/mm^3^. Platelet antigen testing was done on both mother and father, which revealed HPA 1a−/1b+ genotype in mother and HPA 1a+/1b+ heterozygous genotype in the father using the PCR‐sequence‐specific primer (PCR‐SSP) method. Antigen‐matched platelet transfusions were then initiated. Two transfusions were given, sustaining the platelet count at a higher level. A head ultrasound obtained upon admission to NICU was unremarkable for intraventricular hemorrhage. The newborn was discharged from the hospital on Day 6 of life with a platelet count of 108,000/mm^3^ (Figure 1). On outpatient follow‐up at Day 41 of life, repeat laboratory tests revealed a platelet count of 170,000/mm^3^, indicating resolution of thrombocytopenia.

## 3. Case‐2

A second child was born to the same parents 17 months later. This time an appropriate for gestational age (AGA) male at 37 weeks and 5 days gestation and a birth weight of 3572 g was delivered by cesarean section. During this pregnancy, the Gravida 2 Para 1 mother received routine prenatal care and was treated with weekly IVIG at a dose of 1 g/kg/week and daily oral prednisone at a dose of 0.5 mg/kg/day starting at 20 weeks of gestation. The IVIG dose was then increased to 2 g/kg/week at 32 weeks (standard therapy) [2], no FBS was performed. In addition, the mother incidentally received magnesium sulfate and a complete dose of betamethasone at 26 weeks of gestation for presumed preterm labor. The newborn transitioned well after the cesarean section delivery with APGAR scores of 8 and 9 at 1 and 5 min, respectively, and was admitted to NICU for further monitoring. On admission, a complete blood count revealed thrombocytopenia with a platelet count of 24,000/mm^3^. Physical examination was unremarkable except for an isolated finding of hypospadias.

**FIGURE 2:**
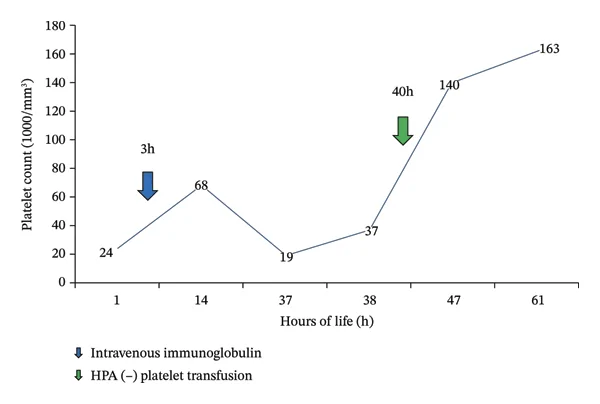
The platelet count of the second sibling throughout his NICU stay in relation to various interventions. The patient received 1 dose of intravenous immunoglobulin and 1 HPA‐negative platelet transfusion.

The newborn received an initial IVIG infusion at a dose of 1 g/kg, resulting in an increase in platelet count to 68,000/mm^3^. Despite this improvement, the platelet count decreased to 37,000/mm^3^ the next day, prompting a transfusion of HPA compatible platelets at a dose of 15 mL/kg over 1 h. Subsequent platelet counts showed improvement, reaching 140,000/mm^3^ six hours posttransfusion and 163,000/mm^3^ the day after (Figure 2). Head ultrasound was performed, which did not reveal any intraventricular hemorrhage. The newborn was discharged from the NICU on the fourth day of life with follow‐up scheduled with a pediatric hematologist. One week after discharge, the platelet count was found to have increased to 260,000/mm^3^.

## 4. Discussion

Severe NAIT defined as platelets less than 25,000/mm^3^ is rare and occurs in 1 in 10,000 live births. Suspicion of NAIT arises when a newborn presents with bleeding problems such as petechiae, ecchymosis, epistaxis, melena, hematuria, or ICH. The incidence of ICH has been reported to be as high as 10%–30% of affected newborn infants, born to mothers with no antenatal treatment [1]. The first pregnancy may be affected, but the diagnosis is revealed inadvertently or only after the birth of a symptomatic infant as exemplified in the current report.

Maternal–fetal incompatibility for HPA‐1a is the prevailing cause of NAIT in families of Caucasian and African ancestry, evident in 85% of cases in which an HPA‐specific antibody is identified. More than 90% of HPA‐1a alloantibodies are made by women positive for the Class II histocompatibility antigen DRB3∗0101, present in about one‐third of the general population [2]. Currently, there are no screening methods available that could be applied to diagnose these cases [5]. Potential screening options for women identified as HPA‐1a–negative, include genotyping for fetal DNA in maternal plasma by cell‐free DNA (cfDNA); however, this is not universally available. The second option is the quantification of maternal serum anti‐HPA. A correlation between the antibody level at the time of delivery and the severity of thrombocytopenia in newborns has been observed. On the other hand, invasive testing such as amniocentesis for fetal HPA status has been abandoned as it poses significant risk to the fetus.

NAIT may recover spontaneously without any intervention in some instances. The optimal method for managing pregnancies at risk of NAIT also remains a subject of ongoing debate, largely due to the absence of randomized, placebo‐controlled trials [6].

A risk stratification of NAIT cases has led to the development of guidelines for antenatal therapy. IVIG administration during pregnancy is currently used in most centers as first line of therapy in NAIT due to its noninvasive nature and effectiveness. The benefit and efficacy of adding prednisone to IVIG is debated and not yet proven [3]. This case series provides additional evidence supporting the combined use of IVIG and prednisone antenatally to enhance outcomes in future pregnancies.

In the context of the second pregnancy, intrapartum treatment was administered according to guidelines, classified as “standard risk pregnancy” where a prior infant exhibited thrombocytopenia without evidence of ICH [7]. The occurrence of ICH in a previous sibling remains the strongest clinical predictor of severe recurrence. The Pacheco/Berkowitz/Bussel risk stratification for NAIT classifies subsequent affected pregnancies into three tiers based on the presence, absence, and timing of ICH in a previously affected sibling [8]. Standard risk is defined as a previously affected sibling having NAIT without ICH. Treatment with IVIG (1 g/kg/week) ± prednisone is typically initiated, around 20 weeks of gestation, with empiric escalation of therapy at 32 weeks, as documented in Case 2.

Furthermore, Case 1 was born at term and was SGA, which may reflect placental dysfunction secondary to anti‐HPA‐1a antibody binding to syncytiotrophoblast cells, resulting in placental inflammation and impaired fetal growth. In contrast, Case 2 was born at term and AGA, possibly suggesting a positive impact of antenatal treatment.

The International Collaborative for Transfusion Medicine Guidelines (ICTMG) recommend women with NAIT in previous pregnancy or sisters of women with NAIT to be referred to fetal–maternal centers for screening and discussion of subsequent pregnancies. In addition, they advise that IVIG should be administered antenatally, starting between 12 and 16 weeks of gestation [9]. Individualized treatment based on the presence of maternal antiplatelet antibodies and the corresponding platelet antigen on fetal cells may aid in identifying subsequent pregnancies where one expects severe thrombocytopenia. This parameter in risk assessment may lead to more selective use of IVIG [10].

The mother in the second pregnancy inadvertently also received magnesium sulfate and antenatal betamethasone at 26 weeks gestation for preterm labor. Maternal magnesium sulfate exposure during pregnancy may alter neonatal platelet function without significantly affecting platelet count [11], and antenatal betamethasone treatment has not been associated with differences in neonatal platelet counts at birth [12].

Regarding mode of delivery, elective caesarean section at 37 weeks of gestation for all women with at risk pregnancies has been suggested, with the aim of reducing trauma. In multiparous women, induction of labor at 38 weeks of gestation with avoidance of instrumental delivery or fetal scalp blood sampling may be an alternative approach [8].

While retrospective studies generally suggest that subsequent pregnancies tend to be more severely affected than the initial one, the lone prospective study focusing on HPA‐1a–immunized pregnant individuals did not corroborate these findings. Within this study, involving 45 pregnant individuals with positive platelet antibody screening and multiple HPA‐1a incompatible pregnancies, important observations emerged. Notably, in instances where the initial pregnancy featured severe thrombocytopenia, neonatal platelet counts in subsequent pregnancies equaled or exceeded those of the initial pregnancy in 29% of cases. During this study, none of the mothers received antenatal IVIG treatment [13].

On the contrary, one notable case highlights the antenatal treatment of the sixth pregnancy of a mother with previous history of severe NAIT, significant for 1 previous stillbirth and 2 newborns with severe thrombocytopenia at birth. During the pregnancy, the mother received IVIG, starting from 31 weeks gestation to delivery at 38 weeks. The newborn had moderate thrombocytopenia at birth, which was resolved after a prophylactic dose of IVIG, without the need for platelet transfusion [14].

The current case report describes two sibling cases of NAIT that required specific treatments of IVIG and HPA genotype compatible platelet transfusions. In this case series, the second child exhibited significantly higher platelet counts compared to the first child, albeit still falling within the severe thrombocytopenia category. However, this second child demonstrated a notably improved clinical presentation, showing no signs of petechiae or ICH on the head ultrasound examination. Additionally, the second sibling had a swifter response to IVIG treatment and platelet transfusion, resulting in a shorter hospitalization duration and expedited return of platelet counts to normal levels (Table 1). This improvement suggests that antenatal intervention using both IVIG and prednisone may play a meaningful role.

**TABLE 1:** Comparison of serial platelet count by day of life in the two siblings showing the platelet count stabilized rapidly in the second case.

Case	Platelet count (per mm^3^)						
1	3000	55,000	12,000	68,000			
		20,000	22,000	66,000	60,000	52,000	108,000
			72,000				

2	24,000	68,000	19,000	163,000			
			37,000				
			140,000				

	Day 0	Day 1	Day 2	Day 3	Day 4	Day 5	Day 6

## 5. Conclusion

In conclusion, a diagnosis of NAIT in this case report was prompted by the presence of clinical symptoms in the first sibling. Following diagnostic evaluation and confirmation, the mother received effective interventions during the subsequent pregnancy resulting in the birth of an infant who was less severely affected. This demonstrates the importance of diagnosis, monitoring and treatment of pregnant women affected by HPA alloimmunization. Additionally, this case emphasizes the need to develop screening methods that can be applied universally to identify pregnancies at risk and determine the best antenatal care.

## Funding

No funding was received for this manuscript.

## Consent

Written informed consent was obtained from parents prior to publication.

## Conflicts of Interest

The authors declare no conflicts of interest.

## Data Availability

The data that support the findings of this study are available on request from the corresponding author. The data are not publicly available due to privacy or ethical restrictions.
